# Prerequisites for self-care actions in individuals with restless legs syndrome—A deductive qualitative analysis based on the COM-B model

**DOI:** 10.1177/13591053251315379

**Published:** 2025-01-31

**Authors:** Elzana Odzakovic, Anna Eliasson, Paula Jansson, Maria Lagerqwist, Bengt Fridlund, Lise-Lotte Jonasson, Martin Ulander, Jonas Lind, Anders Broström

**Affiliations:** 1 Jönköping University, Sweden; 2 Linnaeus University, Sweden; 3 Linköping University Hospital, Sweden; 4 Linköping University, Sweden; 5 County Hospital Ryhov, Sweden; 6 Western Norway University of Applied Sciences, Norway

**Keywords:** self-care, COM-B, counselling, capability, opportunity, motivation, restless legs syndrome, sleep, behaviour, qualitative content analysis

## Abstract

Restless Legs Syndrome (RLS) affects 3% of the world’s population, causing tingling sensations primarily in the legs. Incorporating self-care activities could improve the management of RLS symptoms, yet knowledge about effective self-care actions is limited. This study employs the Capability, Opportunity, and Motivation-Behaviour (COM-B) model to explore self-care behaviours in individuals with RLS, as research in this area is sparse. Qualitative content analysis of interviews with 28 participants with RLS, 26 subcategories emerged, aligning with the COM-B model’s components. The first part, *Capability*, highlighted the importance of being able to be in motion, while the second, *Opportunity* referred to situations where there was a lack of trust and guidance for self-care. The third part, *Motivation*, emphasised the importance of fixed routines of sleep, rest, and activity. These identified prerequisites can inform the development of screening instruments and patient-reported outcome measures to evaluate self-care needs and interventions for individuals with RLS.

## Introduction

Restless Legs Syndrome (RLS) is a prevalent sensory-motor disorder with a circadian rhythm profile (Allen et al., 2014). Two recent meta-analyses, one showing a worldwide prevalence of 3% (Broström et al., 2023), and one showing RLS to cause lower QoL compared to control groups (Broström et al., 2024), highlight the importance of a personalised treatment approach combining updated pharmacological treatment with relevant self-care strategies. The pathophysiology of RLS is not fully understood, but it is believed to be due to iron deficiency in the brain and a dysfunction of dopamine regulation in the central nervous system (Trenkwalder et al., 2018). Symptoms are described as an urge to move the arms and legs, usually associated with a burning, crawling pain, which can lead to anxiety, poor sleep, and reduced quality of life (Holzknecht et al., 2022). In severe cases, all aspects of the life situation are affected (Harrison et al., 2021).

Treatment for RLS consists of pharmacological therapy including iron supplements, alpha-2-delta ligands, dopamine agonists, and opioids (Lv et al., 2021), and/or non-pharmacological treatment (Winkelman et al., 2024). The recommendations are based on three different levels of symptoms (Khachatryan et al., 2022). Even so, the clinician often faces a challenge in achieving satisfactory long-term symptom relief (Lv et al., 2021), partly due to great individual variations of symptoms, but also since some drugs have severe side-effects, for example dopaminergic drugs can lead to augmentation (Garcia-Borreguero, 2018). In light of this, augmentation contradicts the fundamentals of chronic management. The pharmacological treatment itself can lead to a gradual worsening of symptoms, often to a severity not typically seen in the natural course of the disease (Winkelman et al., 2024). Therefore, incorporating self-care strategies becomes crucial, as RLS treatment should prioritize long-term effectiveness. Self-care can serve as an important complementary treatment for both patients with mild and severe symptoms, but further research is needed to guide accurate treatment.

Self-care is performed by the individual and is often essential to cope with a chronic illness (e.g. RLS) and to improve the individual’s quality of life in the long term (Jaarsma et al., 2021). According to Riegel et al. (2019), self-care encompasses three dimensions within their middle-range theory: maintenance (e.g. maintaining physical and emotional stability), monitoring (e.g. observing changes in symptoms), and management (e.g. responding to symptoms). The self-care process is dynamic and varies across individuals and contexts, evolving over time. Effective self-care requires both lay support (personal experience or being a relative of someone affected) (Williamson, 2018) and professional support. While support can enhance self-care, individuals without lay support (e.g. single) can still manage but may encounter additional challenges. Ability, self-confidence, as well as experience and motivation are other important components, where self-care ability for example could be affected by the context (i.e. living conditions) (Riegel et al., 2019). Unfortunately, evidence regarding commonly used and effective self-care actions for RLS does not exist (Chelminiak et al., 2018). Therefore, studies exploring prerequisites for emphasizing factors that facilitate or create barriers to self-care could fill a significant knowledge gap and contribute important knowledge to the healthcare personnel’s toolbox (Fulda et al., 2021).

The Capability, Opportunity, and Motivation-Behaviour model (COM-B) is used to explain sets of activities designed to change specified behaviours patterns (West and Michie, 2020), but it also captures possible causes to understand prerequisites for self-care according to the components included in the model. Identifying prerequisites can guide future interventions (Fulda et al., 2021) and support strategies to improve self-care for individuals with RLS by a holistic care approach (Veerabhadrappa, 2023). Self-care to handle RLS symptoms could be seen as a behaviour. The first part of the COM-B model, capability, includes an individual’s psychological and physical skills, as well as the knowledge necessary to perform the behaviour. Opportunity includes the physical opportunities the environment provides, but also existing social opportunities. External factors that create physical and social opportunities, which either facilitate or create barriers to the behaviour, are also included. More specifically, physical opportunities can be described as the existing and offered resources, while social opportunities can be existing roles and norms within the society. Motivation has two types, reflective and automatic motivation. Reflective motivation includes plans, looking at life, and thoughts the individual has, while automatic reflection includes feelings, needs, and wishes (Michie et al., 2011). The more self-belief and the greater ability the individual have, the more the motivation to implement a behavioural change is seen (West and Michie, 2020).

### Aim

The aim was to determine prerequisites for self-care actions in individuals with RLS based on the COM-B model.

## Method

### Study design

A descriptive deductive design with qualitative content analysis (QCA) (Graneheim & Lundman, 2004) based on the COM-B model was used. QCA was used for interpreting texts where experiences, actions, explicit or implicit rules, codes or power structures are in focus (Graneheim and Lundman, 2004).

### Participants

The Swedish RLS patient organisation, with about 1500 members, was used to establish contact with individuals with RLS. Initially, all members of the organisation were invited to participate in a questionnaire-based survey on RLS with the following inclusion criteria: age over 18 years, having been diagnosed and treated for RLS, ability to speak and understand Swedish, and ability to provide written informed consent. Of the 788 members who gave written informed consent to participate in the survey and who returned questionnaires, 472 (i.e. 60%) expressed their interest in being contacted for a follow-up qualitative in-depth interview. In the next step, a strategic selection of 28 individuals was made from those 472 who accepted to be interviewed. This strategic selection (Robinson, 2014) of participants was made to ensure a clinical diversity in the sample from a RLS perspective. No subsample comparisons were done. Instead, the intention was to create a clinically sound variation in all the mentioned variables. Variation regarding gender, age, education level, cohabitation, comorbidity, severity level of RLS symptoms, and pharmacological treatment was pursued (Supplemental Table 1).

The specific variables were based on the interdisciplinary research teams theoretical and clinical understanding of how RLS is presented in various settings and disease severity levels. The intention was to capture a broad range of clinically sound experiences of prerequisites for self-care.

### Data collection

A written information letter about the qualitative interview study and the data collection procedure (i.e. the actual interview) was sent out to the 28 strategically selected participants, who all agreed to participate. A semi-structured interview guide with open-ended and follow-up questions was developed by the interdisciplinary research team (i.e. physicians, nurses, and sociologists), which had extensive competence regarding the treatment of patients with RLS and QCA. Participants were asked about their experience about their life situation, self-care, and support. Clarification follow-up questions were employed to ensuring a comprehensive exploration and confirming our grasp of the collected information. The interviews were conducted via telephone during June and November 2022 by three members of the interdisciplinary team. The three interviewers had extensive clinical backgrounds as nurses and social workers, along with methodological expertise in QCA and interviewing participants with chronic diseases. After obtaining written and verbal informed consent from the participants, the interviewer provided additional information and answered participant-initiated questions regarding the study. The interviews, lasting 45–90 minutes, were audio-recorded. During this time, COVID-19 was still present but not at its peak prevalence in Sweden.

### Data analysis

Verbatim transcripts of all interviews were produced, resulting in an analysis unit of 571 A4 pages. A latent analysis based on the Graneheim and Lundman (2004) method for deductive QCA was used that is, the analysis was performed based on predetermined categories that is, COM-B model led by the first author. By determining both a manifest and a latent analysis level, an increasingly deep abstraction vis-à-vis the studied phenomenon is achieved. Manifest analysis deals with the surface, visualising components in the text, that is, what the text says, whereas latent analysis concerns underlying meanings, that is, what the text is about (Graneheim and Lundman, 2004). Initially, the interviews were read several times to identify statements (i.e. meaning units) concerning prerequisites for self-care on a manifest level. In the next step, the meaning units were read repeatedly, then compared and clustered on latent level into the three domains of the COM-B model (i.e. capability, opportunity, and motivation) (West and Michie, 2020). During this step, our coding process helped us to systematically assess which factors facilitated or hindered self-care behaviours in line with the domains of the COM-B model. In the last step, the interdisciplinary research team was engaged in discussions to establish a consensus on a category system describing prerequisites for facilitating or impeding self-care.

## Findings

The results are presented according to the three domains in the COM-B model, separated into subcategories describing prerequisites that either facilitate or create barriers to self-care among individuals with RLS (Table 1). Quotations are presented to illustrate the subcategories. All names are presented as pseudonyms.

**Table 1.** Categories and subcategories analysis structure according to the COM-B domains.

COM-B domain	Facilitators	Barriers
Capability		
Physical	• Being able to be in motion • Being able to use relieving body positions • Being able to use alternative physical treatments	• Having the need to be in motion at night causes disturbed sleep • Feeling RLS symptoms made participation in late sedentary social activities difficult
Psychological	• Having the ability to absorb and apply information • Advocating for one’s own needs in care-related decisions • Using distraction to manage discomfort • Daring to go against the body’s signals on selected occasions	• Feeling worried that exercise in the evening worsens the symptoms • Feeling stressed about finding the right drug treatment • Lacking trust in healthcare personnel
Opportunity		
Physical	• Having no other limiting medical diagnoses	• Feeling a lack of medical competence among healthcare personnel • Not getting access to self-care advice from healthcare personnel
Social	• Creating a home environment free from negative societal influences • Having good social support • Having a strong belief in one’s own abilities	• Being forced to refrain from social contexts
Motivation		
Reflected	• Being able to search for pharmacological treatment alternatives yourself • Belief in alternative self-care activities • Having the will to adapt eating habits	• Distrusting the effects of self-care • Prioritising searching for new pharmacological treatment options
Automatic	• Having fixed habits for sleep, rest, and activity • Having fixed times for medication intake	

### Capability

#### Physical factor that facilitates self-care

##### Being able to be in motion

Participants described their ability to be in motion is a prerequisite for self-care, as it impacts their daily functioning. The symptoms were worse in the afternoon and evening. Various actions were used and often adapted to their individual physical abilities and circumstances. In many cases, the participants were in motion until bedtime, engaging in activities for example, walking, jumping, stamping or doing push-ups in front of the TV. They found that the self- care practice of various movements before bedtime, such as stretching and yoga, decreased symptoms. Those who were able to begin the day with a workout felt better overall, while others found relief by staying in bed and pedalling and stretching for a while before getting up to ease symptoms.

I just have to move, that’s it, it’s the only thing that makes it easier. (Lisa, 89 year)

##### Being able to use relieving body positions

Participants described their ability to identify and perform relieving body positions to reduce discomfort and symptoms. Often, they changed position when sitting in an armchair, lying on a sofa, hanging over chair backs, sofa backs and walkers to achieve some relief, and continued watching TV or socialising. Additionally, participants elevated their legs with the help of pillows or held them up against a wall. They sat and dangled their legs back and forth to obtain relief. These positions were mainly used in the evening, as more strenuous exercise was experienced as a trigger that worsened symptoms.

I was hanging on a chair; I was lying across the armchairs. I couldn’t sit. (Bertil, 74 years)

##### Being able to use alternative physical treatments

Being able to apply alternative physical treatments relieved symptoms. Massage, especially on the legs, was carried out either by themselves or with a massage gun. Participants described that the massage gun worked deeper into the muscles and helped the muscles to relax. A vibration plate was expressed by others to intensify the blood flow. Magnetic belts which could give a pain-relieving effect were also used. Additionally, participants expressed how they performed “cupping” and various breathing techniques to manage and reduce their symptoms.

Cupping affects the circulation very much, it does. You push the cup down and then you release it, you get a negative pressure that lifts the skin up, and the connective tissue, and then you pull it back … it works really well. (Elvira, 67 years)

#### Physical barriers to self-care

##### Having the need to be in motion at night causes disturbed sleep

Having to perform movements in the evening and at night led to difficulties both in falling asleep and achieving a full night’s sleep. Participants described how they were often compelled to leave their bed either before falling asleep or during sleep because symptoms, such as spasming arms and legs, and crawling sensations or pain forced them. That led them to get up and walk, do push-ups, or transition from the bed to an armchair to sit and move their legs. Alternatively, others chose to stay in bed and stamp their feet. Push-ups were deemed helpful, especially when experiencing a crawling feeling in the arms, but it was described as inconvenient to perform them in the middle of the night. While participants could go to bed and fall asleep after engaging in self-care activities, the process often needed repetition throughout the night.

If I have a rough night, then I walk a few kilometres. Either I wake up or I get up because I can’t fall asleep. (Bertil, 75 years)

*Feeling RLS symptoms made participation in late sedentary social activities difficult* Feeling symptoms made participation in late social activities difficult, especially if the activity required being sedentary for long periods of time. The social contexts that were challenging included attending lengthy dinners, meetings, or going to a theatre, a movie, or a concert. If the symptoms became too troublesome, participants opted to go home. Those who worked at night described that instead of sitting they walked most of the night. During cultural events, bus or train rides, or air travel, participants preferred seats which allowed an opportunity to stand up and move around to alleviate their symptoms.

What’s hard is, when you sit at long dinners, and just sit. Those who know me are aware of my issues and know I need to stand up, and that’s what I do. It’s not rude to stand up. (Måns, 74 years)

#### Psychological factors that facilitate self-care

##### Having the ability to absorb and apply information

Participants highlighted the importance of acquiring and applying relevant information to manage their symptoms effectively. Understanding their condition and identifying effective strategies helped them tailor self-care to their needs. They actively sought information on symptom management by various sources (e.g. healthcare personnel, scientific papers, or text provided by healthcare organisations) to obtain accurate and up-to-date information. Participants expressed satisfaction with the information provided through their patient organisation and its magazine where updated information and research were shared. Through this source, they also shared experiences and received advice from others who had the disease, as well as contact information to seek advice.

I told my GP what treatment I think we could try because I am quite well-read as I’ve been part of the patient organisation and quite interested and dig into things. (George, 59 years)

##### Advocating for one’s own needs in care-related decisions

Having a good dialogue with knowledgeable healthcare personnel and being involved in care-related decisions was described as important. By advocating for their needs and engaging in open communication, participants aimed to reshape the patient experience and ensure their voices were heard in the decision-making process.

To facilitate this, if they had not met a new physician or were whether the physician knew about their condition, they provided information (e.g. documents, magazines, or books about RLS) that they perceived as important. They also asked for extra time during appointments so that the physician had time to gain a more in-depth understanding of their situation and to enable joint discussions to develop a functional pharmacological treatment. This also allowed the planning of continued care without time pressure and time to find a physician who had knowledge of RLS.

I went to a physician because he was knowledgeable about RLS. You explained your symptoms and he/she understood straight away. (Karin, 66 years)

##### Using distraction to manage discomfort

Various types of distractions were used as facilitator to divert attention from uncomfortable symptoms. These could include quiet activities (e.g. watching TV, reading books or magazines). Activities that required concentration (e.g. solving crosswords, playing musical instruments) significantly reduced symptoms or alleviated them while engaging in the activity. Participants also described how they sat on hard surfaces like a stool or the floor. Scratching their legs with a hairbrush relieved discomfort. Others alternated between hot and cold water in a shower, alternatively choosing only cold water. A similar effect was achieved when standing on wet grass or snow. Warm clothing was avoided by some participants, while others found distraction through heating pads. There were those who travelled to warmer countries to benefit from the heat.

I have hobbies, I do carpentry or embroidery, or do other things, and then, when I focus on something, the symptoms sort of disappear. (Eva, 78 years)

##### Daring to go against the body’s signals on selected occasions

The participants described resilience to unwanted consequences as a facilitator. They dared to participate in certain social activities, even if symptoms were present, and they knew the symptoms would become even worse the following hours, or during the night. They forced themselves to sit up and watch TV together with the family, although they were aware that it would be better to stick to their routines and move around or go to bed. Participants chose to exercise in the evening with friends or colleagues to be social, even though they knew symptoms would increase. Others continued to use snuff, to drink wine with food, or have a few beers with friends, despite knowing that nicotine and alcohol worsened symptoms.

I think it’s fun to have a beer with my friends, but I really must plan these days. ‘How I want to take the hits’, because the symptoms get much, much worse. (Albert, 69 years)

#### Psychological barriers to self-care

##### Feeling worried that exercise in the evening worsens the symptoms

The participants described feeling concerned and anxious. They were worried about exercising late in the afternoon or in the evening because physical activity sometimes increased the discomfort of the symptoms.

After 2 pm in the day I can’t go for walks, I must do it in the morning, because if I go after 2 pm, then my legs hurt terribly in the evening. (Evan, 58 years)

##### Feeling stressed about finding the right drug treatment

The participants described stress about finding the right drug treatment, especially when they were required to try different kinds of drugs and find the appropriate dose tailored to their situation. They faced a lack of clear guidance or recommendations, and they had difficulty understanding their medications. Consequently, they often experimented with the doses themselves. They described that some drugs (e.g. sleeping pills) triggered symptoms instead of relieving them.

I gauge an average value of how I feel, and then …‘well, it will be like this today, yes, but then maybe I’ll try to reduce the dose tomorrow.’ It’s very difficult to manage. (Ali, 63 years)

##### Lacking trust in healthcare personnel

Lack of trust was expressed as a complicating aspect that negatively affected the healthcare personnel’s commitment to treating their disease. Participants described how healthcare personnel could not answer questions about RLS, and they felt that their symptoms were not taken seriously. The lack of trust led participants to feel frustrated and disappointed.

The last time I talked about my symptoms, a physician told me straight to my face, this is a psychological problem. (Inger, 73 years)

### Opportunity

#### Physical factors that facilitate self-care

##### Having no other limiting medical diagnoses

Not having a medical diagnosis that restricted was described as a key facilitator by the participants. Access to safe environments, such as parks, gyms, or well-maintained walking paths, further encouraged physical activities. Others used indoor resources like exercise bikes, pedal machines, or practised yoga and stretching. The availability of these resources, both in public spaces and at home, played an important role. Walking around during conversations, instead of standing still was also highlighted by the participants as a simple but effective way to stay active.

Exercise is very important. You must move, I go to the gym do some jogging. It feels good, being physically active. (Birgitta, 56 years)

#### Physical barriers to self-care

##### Feeling a lack of medical competence among healthcare personnel

The participants felt a lack of RLS-related competence among healthcare personnel. They described this as not receiving adequate help related to their illness and treatment needs, or encountering neurologists who did not know that RLS could also affect the arms. Others pointed out that it would be beneficial if most physicians, nurses, and other healthcare personnel in hospitals and primary care had at least a basic knowledge of RLS. Furthermore, poor knowledge of reference values when taking samples for RLS (e.g. iron) was also mentioned by the participants. Environmental factors such as hospital setting were also noted, as participants feared being confined to a bed in a hospital without the ability to perform self-care or receive their medicines at appropriate times.

And I have to say that lying in hospital, when you lie in bed all the time and don’t get medicine, it must be terrible, with those twitches, because once it starts and I can’t stop it… I tell them I feel bad, it’s hard. I don’t know what to do, I could chop off my legs because it’s so hard.” (Ingrid, 81 years)

##### Not getting access to self-care advice from healthcare personnel

Not getting access to self-care advice hindered participants’ understanding and ability to manage their symptoms effectively. The lack of advice from the healthcare personnel made them seek information from other sources, such as the patient organisation, newspapers, or Facebook groups.

The information I receive regarding self-care and such, it is from the patient organisation. It has meant a lot. (Lennart, 72 years)

#### Social factors that facilitate self-care

##### Creating a home environment free from negative societal influences

Participants emphasized the importance of having a spacious home that facilitated movement, particularly those living with a partner. Many chose to have separate bedrooms, allowing them to move freely in bed and get up without disturbing their partner, which helped alleviate symptoms. Additionally, having two floors provided an opportunity to navigate their space undisturbed, further promoting self-care practices.

We have separate bedrooms because I feel much better if I don’t have to consider disturbing the one lying next to me. I feel at my best when I have my own room and where I can get up and walk around, get up, go to bed, walk a little. (Doris, 58 years)

##### Having good social support

Social support from partners and loved ones, as well as staying within circles of friends who understood the situation, gave participants a sense of security, as they did not mind if there was a need to get up and move around. At work, understanding managers who were generous with flexible working hours and aids (e.g. adjustable desks) were important for the participants. Other examples of support were partners initiating daily activities, or others considering the need to buy theatre tickets for seats at the end of a row so that there was an opportunity to stand up or leave the room if needed.

My husband, he is like a driving force. He says, `Come on, now we get up`. He knows that movement makes it easier. (Amanda, 37 years)

##### Having a strong belief in one’s own abilities

Having a strong belief in one’s own abilities was an important facilitator for managing the disease through self-care, according to the participants. Finding relevant information to learn more about RLS, or which self-care actions suited different times of the day, or when to take prescribed medication was crucial. On the other hand, finding relevant information increased self-confidence and understanding of how self-care could be used to manage symptoms.

I keep practising and keep going and thinking about how to make it as good as possible. (Elsa, 69 years)

#### Social barriers to self-care

##### Being forced to refrain from social contexts

Feeling symptoms made participation in late social activities difficult, especially if the activity required being sedentary for long periods of time. The social contexts that were challenging included attending lengthy dinners, meetings, or going to a theatre, a movie, or a concert. Others refrained from social activities because their patience was impaired by the disease, and they were afraid of losing their temper. There were those who opted out of making close acquaintances and instead chose superficial social contacts, as their mood often changed.

I have to give up a lot in the evenings, I think it’s sad, because I want to go to the theatre, I want to go to a concert, but I don’t dare anymore. (Ingvar, 72 years)

### Motivation

#### Reflected motivation that facilitates self-care

##### Being able to search for pharmacological treatment alternatives yourself

The participants described that they sought pharmacological treatment options through various means, including the Internet, friends, or communication with healthcare personnel. There were those who expressed the need to self-medicate because they had not received helpful prescriptions. Others experimented with prescribed sleeping pills and painkillers obtained from relatives or friends, adjusting doses to find the optimum one for them. Dietary supplements such as magnesium, melatonin, Omega 3, Vitamin D and Vitamin B-12 were also sought.

I read that magnesium and iron could be something, so I tried magnesium this autumn, and together with the iron tablets, it has had a very good effect, so now I do not have many symptoms. (Albertina, 64 years)

##### Belief in alternative self-care activities

Belief in alternative self-care activities (e.g. acupuncture, lymphatic drainage, osteopathy, chiropractic, reflexology, healing, and massage) to relieve or manage their symptoms was described as a facilitator to maintain belief in alternative self-care activities by participants. They expressed a belief that these activities, in combination with pharmacological drugs, alleviated symptoms.

I’ve tried acupuncture, both regular acupuncture and this kind of Chinese acupuncture. (Måns, 68 years)

##### Having the will to adapt eating habits

Having the will to adapt dietary habits was described as a facilitator for self-care by participants. For example, there were those participants who cooked and ate a Mediterranean diet rich in iron, magnesium, and vitamin B, which was thought to have a beneficial effect. Others removed or reduced alcohol, coffee, sugar, and salt, as symptoms worsened with their consumption. Participants also drank stress-relieving tea, as it could promote relaxation and have a calming effect. Drinking lots of water during the day was also experienced to be useful, as well as eating lots of vegetables.

We eat a lot of Mediterranean food, and there are a lot of vegetables such as parsley and spinach because they contain a lot of iron. (Tina, 35 years)

#### Reflected motivation that barriers to self-care

##### Distrusting the effects of self-care

A disbelief in self-care was expressed by the participants since actions they used did not lead to decreased symptoms. This led to increased frustration, anxiety, and stress, consequently, the actions were not used regularly.

Then sometimes I use these, what’s it called, compression stockings, that are tight on my legs, but I don’t know if it helps. (Ylva, 78 years)

##### Prioritising searching for new pharmacological treatment options

The participants expressed that they primarily searched for pharmacological treatment options to relieve their symptoms. Those who did not receive the prescriptions they expected or wanted instead, on their own initiative, tried different kinds of medication given to them by relatives and friends. This, in turn, increased the risk of side-effects and created barriers to managing augmentation.

I use many different painkillers. I’ve tried all kinds of stuff, but nothing really works, but I never stop trying. (Ivar, 77 years)

#### Automatic motivation that facilitates self-care

##### Having fixed habits for sleep, rest, and activity

Having regular routines for sleep, rest, and activity helped participants manage symptoms. These routines created a calm, safe, and structured environment that contributed to symptom relief. Going to bed at the same time every night was described as important and made it easier to fall asleep, as did resting and sleeping for a while at a fixed time during the morning. There were those who also adopted other sleep hygiene actions, such as having a cool, well-aired bedroom, no TV in the bedroom, and trying to relax and unwind before bedtime. Scheduling short breaks for rest helped to reduce fatigue during the day. Participants preferred to engage in training and activity in the morning or forenoon, including walking, cycling, going to the gym, pool training, or specific physiotherapy. Training during the afternoon and evening was habitually avoided as it triggered symptoms.

…you have to be very careful about going to bed at a certain time in the evening and getting up, you must not disturb the partner, then it gets much worse, you are very dependent on routines. (Alice, 69 years)

##### Having fixed times for medication intake

The participants described that they used schedules with fixed times for medication intake. The intake was adjusted to when symptoms occurred with the intention to optimise the effect. They often took their tablets at specific times during the afternoon and evening before bedtime to improve the possibility of sleep. Having a regular routine for their medication intake made it easier to remember when to take them.

I set the clock, I’m very careful with this. Then at ten fifteen in the evening I take a Lyrica and then at half past ten I take a full 0.18 mg of Sifrol^®^ [pramipexole]. Before I go to bed, then I take a Citodone^®^ [paracetamol+oxycodone]. (Otto, 71 years)

## Discussion

This study which aimed to determine prerequisites for self-care actions in individuals with RLS based on the COM-B model is, to the best of our knowledge, the first of its kind. It identifies a wide range of factors that might act as facilitators or barriers to self-care activities, which will be discussed in relation to the components of the COM-B model: capability, opportunity, and motivation (West and Michie, 2020).

### Capability

A novel element of this study was our focus on capability-related physical and psychological prerequisites that were identified as facilitating or hindering self-care for physical and emotional stability. We found that an important facilitator for maintaining self-care for the purpose of preserving health or enhancing well-being (Riegel et al., 2019) was the consistent practice of exercise. This suggests that individuals with RLS are more attuned to bodily cues, including recognised symptoms, compared to other self-care activities, thereby constituting one method of self-management for RLS symptoms, which is in line with previous research (Iovino et al., 2021). In this study, we found that there are individuals who actively follow their self-care routines, while others may follow them passively. All participants possess the capability and knowledge to perform their self-care activities. An accepting attitude toward one’s self-care plan and understanding when RLS symptoms occur and worsen, as well as how to manage them (Björk et al., 2023), such as through exercise, can enhance the capability and motivation for self-care management and self-care monitoring.

Self-care management refers, according to Riegel et al. (2019), to maintenance, monitoring, and management of recognised symptoms or signs. In the current study, feeling RLS symptoms, and not being able manage them, was described as a barrier, since it made participation in late sedentary social activities, especially in unfamiliar social contexts, difficult. On the other hand, seeking psychological distractions, such as diverting attention from RLS symptoms by focusing on alternative stimuli or activities requiring concentration to alleviate pain, were examples of self-care management that could be helpful. These findings are consistent with those of Hestmann et al. (2023), who reported reduced pain levels with distraction techniques in individuals with chronic pain. Other psychological distractions, such Internet-based cognitive behavioural therapy (ICBT), i.e. therapy provided through a computer or a mobile device, are nowadays a common solution to improve the accessibility of cognitive interventions (Kumar et al., 2017). Recent small-scale studies have shown promising results for CBT in addressing sleep issues related to RLS (Song et al., 2020). If adapted for RLS and delivered online, ICBT could provide the individual with capabilities to maintain, monitor, and manage self-care over time (Riegel et al., 2019).

### Opportunity

The second novel aspect of this study was the identification of opportunity-related physical and psychological prerequisites that either facilitated or hindered self-care monitoring. We found that established routines for sleep, rest, activity, and medication intake were essential for facilitating self-care maintenance. These routines, which could be seen as physical prerequisites, provided a sense of security (i.e. a psychological prerequisite) which helped with monitoring and managing RLS symptoms over time. Self-care maintenance (i.e. the individual’s intention to maintain physical and emotional stability) seemed to increase with longer disease duration among individuals in this study. These results are consistent with previous studies, where individuals with heart failure who had lived with the disease for a long time exhibited higher self-care maintenance, suggesting that they tended to acquire more self-care knowledge or skills as their disease duration increased. To enhance and provide opportunities for self-care monitoring for individuals with RLS, fixed routines and schedules should be emphasised as these provide a supportive environment that might reduce symptoms and facilitate self-care monitoring.

We identified having a home environment that allows movement and good social support as social facilitators that led to a sense of security and understanding regarding RLS. Additionally, belief in oneself and involvement in care-related decisions were prerequisites that facilitated self-care monitoring. These findings are consistent with previous studies that have examined self-care for individuals with chronic illness. These studies reported that self-care management and monitoring increased with the quality of social relationships among family and friends (Iovino et al., 2021). According to Riegel et al. (2019), the three elements of self-care (i.e. maintenance, monitoring, and management) can be separate, but also linked because behaviours cannot change without self-recognition. In our study, individuals had good relationships within their social network. Therefore, the effect of social opportunities on self-care management and monitoring suggests that other people, such as friends and family, impact the ability of individuals with RLS to engage in self-care actions.

Furthermore, this study revealed that a lack of trust in healthcare personnel posed a significant challenge to self-care management, monitoring, and maintenance. The lack of trust might mirror the findings presented by Lv et al. (2021) that they state that individuals with RLS often do not receive a diagnosis, potentially due to varying symptom descriptions (Holzknecht et al., 2022), which are difficult to interpret for healthcare personnel. This can result in individuals receiving treatment for conditions they do not have, leading to a reduced quality of life (Broström et al., 2024) and unnecessary side effects. A previous study has shown that empathetic understanding is crucial in building trust between patients and healthcare personnel, and for fostering a sense of security (Moudatsou et al., 2020). Rigel et al. (2012) also stressed the importance of self-care monitoring in achieving treatment goals, alongside receiving relevant and understandable self-care advice from healthcare personnel. The opportunity-related prerequisites identified in the current study could be seen as an aid for healthcare personnel in providing information on RLS-related self-care activities to those in need of such advice.

### Motivation

The third novel result of this study was its revelation of the various self-care maintenance activities described to alleviate RLS symptoms, especially when combined with pharmacological treatment. According to Riegel et al. (2019), belief in alternative self-care activities is a core aspect of self-care management, maintenance, and monitoring. We found that the individuals searched for new pharmacological treatment options to be able to manage their symptoms. The fact that the participants had to make their own attempts to find their ideal individual dosage of medications increases the risk of augmentation (Garcia-Borreguero et al., 2018). Yeh et al. (2023) claimed that it would be worthwhile for individuals with RLS to add another treatment option, where the risk of augmentation has not been confirmed. Adequate medications are needed for symptom relief (Chenini et al., 2023) , but self-care management might be as important, if not more important, as they can be individually adapted based on the individual’s conditions. We found belief in alternative self-care activities as something that facilitated the management of an individual’s symptoms. Therefore, accessible information about what self-care actions you can do yourself, should be provided since it could increase motivation.

Apart from pharmacological treatments, the willingness to adapt one’s eating habits greatly facilitated the self-care management of symptoms. Participants adjusted their diets to include elements such as the Mediterranean diet, which is rich in iron, magnesium, and vitamin B, tailoring these elements to their RLS condition. Brandão et al. (2023) found that diets high in fibre, such as the Mediterranean diet, were associated with better sleep quality, whereas diets high in fat and sugar were linked to poorer sleep. Additionally, according to Cederberg et al. (2023), individuals with RLS often suffer from vitamin D deficiency. Evidence-based recommendations has also reported association between higher levels of magnesium levels and the severity of RLS (Workinger et al., 2018) . We found that individuals avoided foods high in sugar, salt, caffeine, and alcohol, as these could exacerbate symptoms. Batool-Anwar et al. (2016) underscored that adopting a healthy lifestyle reduces the risk of RLS. Living healthily might reduce the risk of RLS, regardless of gender.

Moreover, we found that a lack of trust in self-care maintenance posed a challenge for participants in alleviating their symptoms as they primarily searched for new pharmacological treatment options. Riegel et al. (2019) emphasise that effective self-care maintenance requires trust in others, but also in oneself. Frustration arose when participants did not experience the desired effects from their self-care efforts. This distrust may stem from a lack of knowledge and insufficient time practising self-care for noticeable effects. Merely seeking new pharmacological treatments made self-care management difficult for participants. Hence, further research should explore the impact of self-strategies from a patient perspective and identify factors that hinder self-care maintenance for individuals with RLS.

### Strengths and limitations

The study has several strengths and limitations. A limitation includes selecting participants from a patient organisation, which may have influenced their level of engagement levels. The participants might have been more knowledgeable and proactive in managing their condition, using a wider range of self-care activities, potentially affecting the findings. However, despite being drawn from a patient organisation, the dataset, including 28 strategically selected participants, showed substantial variation, enhancing the study’s clinical credibility and transferability (Lincoln and Guba, 1985). Telephone interviews, though lacking visual cues, facilitated a relaxed environment for discussing sensitive information. Potential bias from the interdisciplinary research team was addressed through extensive discussions, thereby enhancing data dependability and confirmability. A strength is that participants felt comfortable and were able to freely disclose sensitive information about their RLS diagnosis. Thirdly, a limitation could be the potential influence of the three interdisciplinary research team members who conducted the interviews. However, the risk of discrepancies in our data collection was mitigated by extensive discussions in the interdisciplinary team, which allowed for different perspectives on the issue under study and increased dependability and confirmability, which is a strength to this study. Lastly, the deductive approach was grounded in the COM-B theory (West and Michie, 2020). A strength of using the COM-B model lies in its ability to identify facilitators and barriers that could serve as prerequisites for self-care in managing RLS. However, it is important to recognize that while the COM-B model is widely used in behavioural sciences, it has received criticisms for not accounting for environmental factors, such as networks. Our analysis identified individual environmental and social factors affecting self-care, but did not address broader group or societal factors, a limitation of using this model. Despite this, a strength of our analysis was the rich variation and detailed descriptions of the COM-B model components, adding depth and increasing the credibility and transferability (Lincoln and Guba, 1985) of our findings. These findings can inform the development of screening instruments and initiate discussions about self-care management in conversations with patients with RLS (Odzakovic et al., 2024).

### Conclusion

This study has contributed by presenting a variety of prerequisites for self-care actions related to capability, opportunity, and motivation to be used by individuals with RLS. The study has also highlighted specific factors that facilitated self-care actions or made them harder. The knowledge could be used by healthcare personnel to guide them when giving self-care advice to individuals with RLS. To enhance self-care actions effectively, it is essential to incorporate new knowledge. Holistic and person-centred self-care interventions addressing physiological, psychological, and social needs, should be developed. These interventions can assist healthcare personnel in delivering high-quality care.

## Supplemental Material

sj-docx-1-hpq-10.1177_13591053251315379 – Supplemental material for Prerequisites for self-care actions in individuals with restless legs syndrome—A deductive qualitative analysis based on the COM-B model Supplemental material, sj-docx-1-hpq-10.1177_13591053251315379 for Prerequisites for self-care actions in individuals with restless legs syndrome—A deductive qualitative analysis based on the COM-B model by Elzana Odzakovic, Anna Eliasson, Paula Jansson, Maria Lagerqwist, Bengt Fridlund, Lise-Lotte Jonasson, Martin Ulander, Jonas Lind and Anders Broström in Journal of Health Psychology
