# Supplementary material for: Prerequisites for self-care actions in individuals with restless legs syndrome—A deductive qualitative analysis based on the COM-B model
Source: J Health Psychol. 2025 Jan 31;30(13):4059–74. doi: 10.1177/13591053251315379 (PMC12618701; doi:10.1177/13591053251315379)
Supplement: sj-docx-1-hpq-10.1177_13591053251315379 – Supplemental material for Prerequisites for self-care actions in individuals with restless legs syndrome—A deductive qualitative analysis based on the COM-B model [file sj-docx-1-hpq-10.1177_13591053251315379.docx]

**Table for supplement online.** Socio-demographic and situational data of the participants with Restless legs syndrome (RLS) (N=28)

**Variables Value**

**Gender**, female, n (%) 16 (57)

**Age** (years), mean (range) 67.6 (39 – 89)

**Educational level**, n (%)

9 years or below 3 (11)

12–13 years 11 (39)

University 14 (50)

**Civil status**, n (%)

Married/Living together 20 (71)

Unmarried and living alone 3 (11)

Divorced/widower and living alone 5 (18)

**Smoking**, n (%)

Yes, n (%) 1 (4)

**Alcohol**, n (%)

Never uses alcohol 8 (29)

Uses alcohol 2-3 times or more/week 11 (39)

**Comorbidity**, n (%)

Renal disease 0 (0)

Parkinson’s disease 0 (0)

Multiple sclerosis 0 (0)

Migraine 3 (11)

Iron deficiency 4 (14)

**Pharmacological treatment**, n (%)

Dopamine agonists 22 (80)

Opioids 8 (29)

α2δ Ligands 7 (25)

Dopa/derivates 5 (20)

Iron supplement 3 (11)
